# Inferring the Genomic Landscape of Recombination Rate Variation in European Aspen (*Populus tremula*)

**DOI:** 10.1534/g3.119.400504

**Published:** 2019-11-19

**Authors:** Rami-Petteri Apuli, Carolina Bernhardsson, Bastian Schiffthaler, Kathryn M. Robinson, Stefan Jansson, Nathaniel R. Street, Pär K. Ingvarsson

**Affiliations:** * Linnean Centre for Plant Biology, Department of Plant Biology, Uppsala BioCenter, Swedish University of Agricultural Science, Uppsala, Sweden,; † Umeå Plant Science Centre, Department of Ecology and Environmental Science, and; ‡ Umeå Plant Science Centre, Department of Plant Physiology, Umeå University, Umeå, Sweden

**Keywords:** linkage disequilibrium, linkage map, linked selection, methylation, nucleotide diversity, recombination

## Abstract

The rate of meiotic recombination is one of the central factors determining genome-wide levels of linkage disequilibrium which has important consequences for the efficiency of natural selection and for the dissection of quantitative traits. Here we present a new, high-resolution linkage map for *Populus tremula* that we use to anchor approximately two thirds of the *P. tremula* draft genome assembly on to the expected 19 chromosomes, providing us with the first chromosome-scale assembly for *P. tremula* (Table 2). We then use this resource to estimate variation in recombination rates across the *P. tremula* genome and compare these results to recombination rates based on linkage disequilibrium in a large number of unrelated individuals. We also assess how variation in recombination rates is associated with a number of genomic features, such as gene density, repeat density and methylation levels. We find that recombination rates obtained from the two methods largely agree, although the LD-based method identifies a number of genomic regions with very high recombination rates that the map-based method fails to detect. Linkage map and LD-based estimates of recombination rates are positively correlated and show similar correlations with other genomic features, showing that both methods can accurately infer recombination rate variation across the genome. Recombination rates are positively correlated with gene density and negatively correlated with repeat density and methylation levels, suggesting that recombination is largely directed toward gene regions in *P. tremula*.

Meiotic recombination (hereafter recombination) is an important evolutionary force that directly alters levels of linkage disequilibrium (*e.g.*, Wright 1931). Recombination therefore has important consequences for how effective natural selection is at removing deleterious mutations (Felsenstein 1974), increasing the frequency of beneficial mutations (Barton 1995) and for determining the resolution of association mapping for the dissection of quantitative traits (Nordborg and Weigel 2008). Recombination rates are known to vary between species, among individuals within species and even among different regions in a genome (Nachman 2002).

Local recombination rates have been shown to be positively correlated with neutral genetic diversity across a wide range of organisms (reviewed in Nachman 2002). One possible explanation for such an association is that cross-over events and/or associated processes, such as gene conversion and double-strand break repair, have direct mutagenic effects and thus act to increase nucleotide polymorphism (*e.g.*, Kulathinal *et al.* 2008). An alternate explanation is that natural selection has indirect effects on sites linked to a site under selection and therefore also acts to reduce diversity on these sites (Begun and Aquadro 1992). Since recombination breaks down linkage disequilibrium, areas of high recombination are characterized by a rapid decay of linkage disequilibrium and linked selection will hence impact fewer sites in the vicinity of a selected site in these regions (Begun and Aquadro 1992). Conversely, in areas of low recombination rates, linkage disequilibrium will be extensive and indirect selection will impact a larger genomic region. Variation in recombination rates across the genome will generate an association between recombination and sequence diversity. Local variation in recombination rates is therefore an important factor for understanding how natural or artificial selection shapes sequence diversity across the genome of an organism. Recombination rates are also known to be associated with a number of different genomic features, such as gene density, repeat density, and cytosine methylation, although the magnitude and direction of these associations are still under debate. Recombination rates have been shown to be both positively and negatively correlated with gene density (positively: *e.g.*, Wang *et al.* 2016, negatively: *e.g.*, Giraut *et al.* 2011), GC-content (positively: Kim *et al.* 2007, negatively: Giraut *et al.* 2011), repeat density and methylation levels (positively: *e.g.*, Rodgers-Melnick *et al.* 2015, negatively: Giraut *et al.* 2011). Characterizing associations between recombination rates and various genomic features at the genus or species level is thus important to avoid making incorrect assumptions about the strength and/or direction of these associations.

Traditionally, recombination rates have been estimated from the relationship of marker positions in linkage maps (Stapley *et al.* 2017) and more recently recombination rates have also been linked to physical regions of a genome through whole genome sequencing (Nachman 2002). However, producing linkage maps is time consuming and may even be infeasible in some species as it in many cases requires controlled crossing of known parents and the establishment of a large segregating progeny population (Stapley *et al.* 2017). Therefore, methods have been developed that infer recombination rates from linkage disequilibrium (LD) between segregating polymorphisms in individuals sampled from natural populations (*e.g.*, McVean *et al.* 2004, Chan *et al.* 2012). Due to the relative ease of obtaining sequence information with modern sequencing methods even from wild populations, these LD-based methods for estimating recombination rates have been widely employed (*e.g.*, McVean *et al.* 2004, Kulathinal *et al.* 2008 Silva-Junior and Grattapaglia 2015, Wang *et al.* 2016, Booker *et al.* 2017). Detailed knowledge of local variation in recombination rates can be used to infer the action of linked selection by establishing a correlation between the levels of nucleotide diversity and recombination rates across the genome of an organism (McVean *et al.* 2004, Chan *et al.* 2012). Using polymorphism data to infer recombination rates and then using these inferred recombination rates to explain variation in genetic diversity could be problematic, but simulations and studies performed using well-established animal model species such as *Mus musculus* (Booker *et al.* 2017) and in a number of *Drosophila* species (Kulathinal *et al.* 2008, Chan *et al.* 2012) suggest that indirect methods for estimating recombination rates are not strongly affected by natural selection. However, comparisons of LD-based and genetic linkage map-based methods for estimating recombination rates are not readily available in plant species. Genome structure, and in particular local rates of recombination, show large scale differences between plants and animals (Haenel *et al.* 2018), and it would therefore also be valuable to assess how well indirect methods for inferring recombination rates perform in plants.

The genus *Populus* has emerged as an important model system for forest genetics due to its rapid growth rate, ability to generate natural clones and a manageable genome size of ca. 480 Mbp distributed across a haploid set of 19 (2*n* = 38) chromosomes (Taylor 2002, Lin *et al.* 2018). Furthermore, both large and small scale synteny is highly conserved across species in the genus, enabling the transfer of genetic resources between species within the genus (Jansson and Douglas 2007). Further interest in *Populus* has been spurred by their economical (*e.g.*, Taylor 2002) and ecological importance (*e.g.*, Kouki *et al.* 2004) and over the past two decades a growing number of the ca. 40 species in the genus have been fully sequenced, including *Populus trichocarpa* (Black cottonwood) (Tuskan *et al.* 2006), *P. euphratica* (Ma *et al.* 2013) and *P. tremula* (European aspen) (Lin *et al.* 2018). Linkage maps have also been produced for many of the species in the genus (*e.g.*, Paolucci *et al.* 2010, Tong *et al.* 2016), including *Populus tremula* (Zhigunov *et al.* 2017) but these maps have generally been relatively coarse, utilizing a few hundred up to a few thousand markers and typically employing mapping populations consisting of fewer than 300 progenies. Consequently, many of these maps have failed to resolve the expected 19 linkage groups typical for the genus and there is thus a need for developing a high-resolution, fine-scale linkage maps for the whole genus. *P. tremula* is a species of special interest within the genus as it has the largest distribution of any tree species in Eurasia, spanning from Spain and Scotland in the west to pacific China and Russia in the east, Iceland and northern Scandinavia in the north to northern Africa and southern China in the south (Luquez *et al.* 2008). Such extensive geographic distribution means *P. tremula* has adapted to a great variety of different environments, making it a promising species for studying the effects of spatially varying selection and adaptation (*e.g.*, Farmer 1996, Luquez *et al.* 2008, Wang *et al.* 2018). Here we present a newly developed, fine-scale genetic map for *Populus tremula* and use this map to anchor scaffolds from the current draft genome assembly of *P. tremula* (Potra v1.1, Lin *et al.* 2018) to chromosomes. We then use this new resource to estimate local variation in recombination rates and use this to assess the correlation with recombination rates inferred from data on linkage disequilibrium in sample of unrelated individuals. We assess how different genomic features, such as gene density, repeat content and methylation levels are associated with estimates of local recombination rates. These results provide a valuable resource for enhancing our understanding of genome evolution and the recombination landscape in *Populus* and will also further facilitate the identification of loci controlling quantitative traits of ecological and economic value.

## Material and methods

### Plant material

In 2013, a controlled F_1_ cross was performed between two unrelated *P. tremula* individuals (UmAsp349.2 x UmAsp229.1) from the Umeå Aspen (UmAsp) collection that consists of c. 300 individuals collected in the vicinity of Umeå in northern Sweden (Fracheboud *et al.* 2009). This cross yielded 764 full sib progenies that were planted and monitored in a common garden at the Forestry Research Institute of Sweden’s research station in Sävar, 20 km north-east of Umeå (63.9N 20.5E). In addition, we utilized SNP data for 94 individuals of *P. tremula* belonging to the SwAsp collection that consists of 116 individuals sampled from 12 sites across Sweden (6-10 individuals per site, Luquez *et al.* 2008) displaying no population structure (Wang *et al.* 2018). The SNP data has previously been described in Wang *et al.* (2018) and consists of 4,425,109 SNPs with a minor allele frequency exceeding 5%.

### DNA extraction and sequence capture

In 2015 leaf samples were collected from all progenies of the F_1_ cross. DNA was extracted using the Qiagen Plant Mini kit according to manufacturer guidelines and sent to Rapid Genomics (http://www.rapid-genomics.com) for genotyping using sequence capture probes. The probe set contain 45,923 probes of 120 bases each that were designed to target unique genic regions in the v1.1 *P. tremula* genome assembly (Lin *et al.* 2018), as well as an additional 70 probes that were designed to specifically target the putative sex determination region on chromosome 19 of the *P. trichocarpa* genome assembly v3.0 (https://phytozome.jgi.doe.gov/pz/portal.html). Parents and all offspring were subjected to sequence capture and subsequently sequenced on an Illumina HighSeq 2000 platform using paired-end (2x100bp) sequencing to an average depth of 15x per sample. All sequence capture data were delivered from Rapid Genomics in the spring of 2016. In addition, the two parents of the F_1_ cross were whole-genome re-sequenced to an average depth of 15x on an Illumina HiSeq 2500 platform with paired-end sequencing (2x150 bp) at the National Genomics Infrastructure at the Science for Life Laboratory in Stockholm, Sweden.

All raw sequencing reads were mapped against the complete *P. tremula* v.1.1 reference genome using BWA-MEM v.0.7.12 (Li and Durbin 2009) using default parameters. Following read mapping, PCR duplicates were marked using Picard (http://broadinstitute.github.io/picard/) and local realignment around indels was performed using GATK RealignerTargetCreator and IndelRealigner (McKenna *et al.* 2010; DePristo *et al.* 2011). Genotyping was performed using GATK HaplotypeCaller (version 3.4-46, (DePristo *et al.* 2011; Van der Auwera *et al.* 2013) with a diploid ploidy setting and gVCF output format. CombineGVCFs was then run on batches of ∼200 gVCFs to hierarchically merge samples into a single gVCF and a final SNP call was performed using GenotypeGVCFs jointly on the combined gVCF file, using default read mapping filters.

### Construction of a high-density linkage map

Linkage maps were built separately for the two parents using a pseudo-testcross strategy (Grattapaglia & Sederoff 1994) by employing bi-allelic SNPs that were segregating according to Mendelian expectations. Pairwise estimates of recombination frequency were calculated between all markers and marker pairs showing no evidence for recombination were collected into bins and one representative marker was used for map construction. Markers were grouped into linkage groups (LGs) using a LOD threshold of 12 and ordered using the Kosambi mapping function as implemented in the BatchMap software (Schiffthaler *et al.* 2017). AllMaps (Tang *et al.* 2015) was used to create physical chromosomes from the *P. tremula* genome assembly v.1.1 (Lin *et al.* 2018) and the male and female genetic maps, with equal weight given to the two maps. For more details about the construction of the genetic and physical maps, please refer to Supplementary Methods.

### Genetic map and linkage disequilibrium-based recombination rates

The genetic and physical maps for all 19 *P. tremula* chromosomes were read into MareyMap (Rezvoy *et al.* 2007, https://cran.r-project.org/web/packages/MareyMap) and the ‘sliding window’ method in MareyMap was used to estimate recombination rate in windows of 1 Mbp with a step size of 250 kbp. We only used windows with at least 8 SNPs in order to avoid regions with large gaps being assigned recombination values.

To generate a LD-based recombination map we used LDhelmet v.1.10 (Chan *et al.* 2012) with a random subset of 25 diploid individuals (*i.e.*, 50 haplotypes) from the SwAsp data set (Wang *et al.* 2018). We used the default values from the LDhelmet manual as *Populus tremula* has similar levels of nucleotide polymorphism and extent of linkage disequilibrium as *Drosophila melanogaster*, on which the default settings in LDhelmet are based upon (Wang *et al.* 2016). As LDhelmet outputs estimates of recombination in units of ρ/bp whereas the genetic map is in units of cM, we converted the LDhelmet results to cM distances following the method outlined in Booker *et al.* (2017). We did this to be able to make comparisons between the recombination rates estimated using the two methods. The conversion assumes that the physical size of a chromosome is constant for the two methods so that the cumulative genetic distance in either cM or ρ should be the same but on different scales. The cumulative ρ was calculated by multiplying the ρ/bp estimates with the distance in bp between the adjacent SNP’s and then summed across chromosomes. Knowing the cumulative ρ and corresponding cM-values, it is possible to derive a ‘scaling factor’ to calculate cM values from the corresponding ρ values. The resulting cM values were read into MareyMap and recombination rates were estimated as described earlier for the genetic map-based recombination map. More details on the estimation of recombination rates from the genetic maps can be found in Supplementary Methods.

### Correlation of recombination rate estimates, genetic correlates of recombination rate and model of recombination rate

We compared recombination rates inferred from the consensus genetic map or from the sequence data by calculating Spearman’s rank correlations (hereafter correlations) across 1 Mbp windows. We also assessed correlations between the two recombination rates and a number of genomic features, including gene density, repeat density, GC-content, substitution density, neutral diversity (π) and methylation.

Gene and repeat density were estimated using bedtools (Quinlan 2014) based on the annotation for the v1.1 *P. tremula* genome (Lin *et al.* 2018, ftp://plantgenie.org/Data/PopGenIE/Populus_tremula/v1.1/gff3/). GC-content was calculated from the genome FASTA using an in-house developed awk script (modified from: https://www.biostars.org/p/70167/#70172). The original script was modified to have window functionality across a FASTA sequence and to take into consideration sequence gaps. Windows with more than 80% gaps (N) were discarded to avoid biased results.

Substitutions relative to *P. trichocarpa* were estimated from a vcf-file with SNP-calls for a single *Populus trichocarpa* individual mapped against the *P. tremula* reference genome v1.1. Comparisons of putative substitutions were then made against a list of SNP positions from the 94 SwAsp *P. tremula* data set. Substitutions relative to *P. tremuloides* were inferred in a similar way based on SNP calls from five *P. tremuloides* individuals mapped against the *P. tremula* reference genome v1.2 (Lin *et al.* 2018). Files containing old, new and the total number of substitutions were used to calculate substitution densities in windows across the genome. Neutral genetic diversity (π) was estimated using vcftools and only fourfold degenerate sites or intergenic sites located at least 2 kbp from an annotated gene. For nucleotide diversity calculations, data for all 94 SwAsp samples were used.

Methylation levels were estimated using bisulfite sequencing data from six SwAsp individuals that were bisulfite sequenced using two biological replicates per individual. Samples were sequenced using paired-end (2x150) sequencing on an Illumina HiSeq X at the National Genomics Infrastructure facility at Science for Life Laboratory in Uppsala, Sweden. Methylation levels were estimated using the Bismark tools (Krueger and Andrews 2011, https://www.bioinformatics.babraham.ac.uk/projects/bismark/). Following trimming and quality control, sequence reads were mapped against polymorphism-substituted versions of the *P. tremula* v1.1 assembly (Lin *et al.* 2018) for each individual sample. Following read mapping BAM files were deduplicated to remove optical duplicates. Methylation levels were then extracted separately for the different methylation contexts (GpG, CHG and CHH) and average values were calculated across 1 Mbp window using a step size of 250 kbp across the *P. tremula* genome. Further details on the handling of the methylation sequencing data can be found in Supplementary Methods. Correlations between the two recombination maps and between the recombination maps and the various genomic features were calculated in R (R Core Team 2018). We also assessed the independent effects of different genomic features on the two recombination rate estimates using multiple regression.

### Data availability

The raw sequencing reads used for the construction of the genetic map and raw bisulfate sequencing reads are available from ENA under study number PRJEB34662 (https://www.ebi.ac.uk/ena/data/view/PRJEB34662). BatchMap input files for the female and male genetic maps, the two component maps and the consensus map files as well as all processed files of the bisulfate sequencing data are available from zenodo.org under DOI number 10.5281/zenodo.3257544 (https://doi.org/10.5281/zenodo.3257544). Additional scripts and files used for the analyses are available at https://github.com/parkingvarsson/recombination_rate_variation. All SNP data used for population genomic analyses is already publicly available through ENA under accession number PRJNA297202 (https://www.ebi.ac.uk/ena/data/view/PRJNA297202). A filtered VCF files with final SNP data are available to download from zenodo.org under DOI number 10.5281/zenodo.3546316 (https://doi.org/10.5281/zenodo.3546316). Supplemental material available at figshare: https://doi.org/10.25387/g3.8481641.

## Results

### P. tremula linkage maps

14,598 unique markers in the female map and 13,997 unique markers in the male map were distributed across 3,861 and 3,710 scaffolds, respectively. Markers in both parental framework maps grouped into 19 linkage groups (LGs), corresponding to the haploid number of chromosomes in *Populus* (Table 1). Among the mapped scaffolds, 19 scaffolds contained markers that mapped to different positions within the same LG, but that were more than 20 cM apart (Supplemental Material, Figure S3) and 340 scaffolds contained markers that mapped to two or more LGs (Figures S2 and S4). These scaffolds were split according to criteria described in Supplementary methods. Additionally, there were 49 scaffolds split within gene models (Figure S5). Two ambiguous markers in scaffold Potra001073 were removed. After splitting 14,596 (12,900 binned) and 13,996 (12,382 binned) markers from 4,184 and 4,011 scaffolds remained. These markers spanned 4072.72 cM and 4053.68 cM for the female and male framework maps, respectively. The parental maps were used to produce a consensus genetic map consisting of 19,519 markers derived from 4,761 scaffolds spanning 4059.00 cM (Table 1). Linkage groups (LG) were assigned to the corresponding *P. trichocarpa* homologs through synteny assessment (Table 1, Figure S1).

**Table 1:** Summary of female male and consensus linkage maps for each chromosome

Chr	LG	Female			Male			Consensus	
		Probe markers	Bin markers	Size (cM)	Probe markers	Bin markers	Size (cM)	Probe markers	Size (cM)
1	6	1,784	1,573	498.0	1,737	1,542	499.3	2,417	498.8
2	3	1,054	924	266.3	1,028	904	268.3	1,407	266.3
3	8	826	736	229.9	790	695	235.3	1,117	235.2
4	1	813	728	217.2	835	736	263.0	1,128	220.5
5	7	965	842	284.5	947	836	270.6	1,293	271.9
6	14	1,241	1,108	311.1	1,116	993	294.3	1,602	296.4
7	18	572	497	161.9	501	450	155.1	765	155.5
8	12	895	781	227.4	824	726	211.0	117	215.5
9	2	697	617	171.5	640	578	171.6	879	172.2
10	4	1,026	901	279.0	1,015	879	270.8	1,388	273.7
11	16	515	460	181.4	541	480	180.7	724	182.0
12	17	515	452	142.4	486	429	140.7	678	143.3
13	11	586	530	173.7	608	531	176.4	822	178.7
14	5	733	655	194.3	702	609	182.0	983	194.3
15	10	553	505	145.8	593	532	157.9	777	159.2
16	19	383	334	145.8	220	195	125.8	430	145.4
17	15	488	433	155.5	447	394	148.9	647	149.4
18	13	591	502	156.3	592	531	166.2	792	165.5
19	9	361	322	130.6	375	342	135.8	500	135.8
Total		14,598	12,900	4072.7	13,997	12,382	4053.7	19,519	4059.0

### Physical assembly Potra v1.2

**Figure 1:**
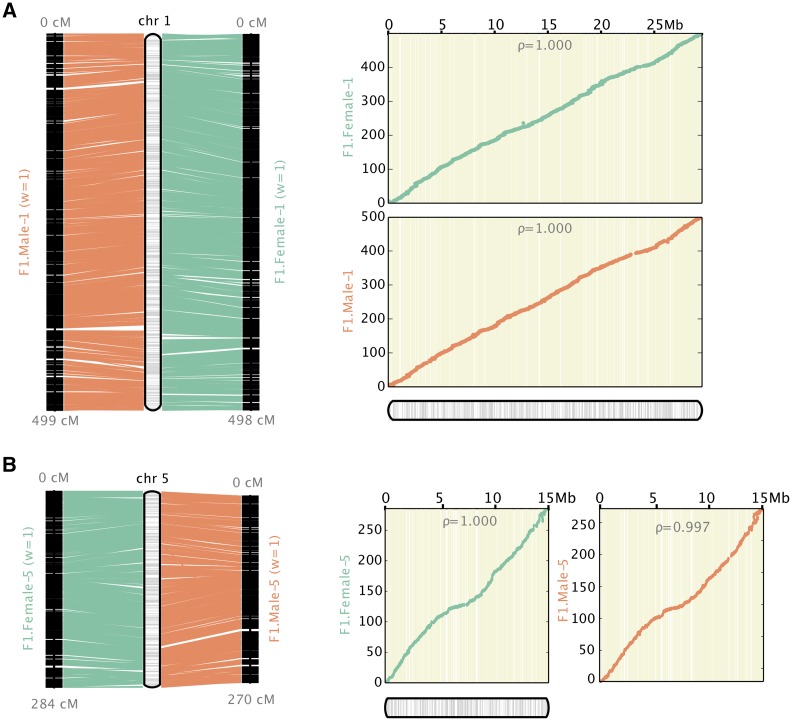
Genetic maps and resulting physical map created by Allmaps for Chr1 and Chr5. The left panel shows the marker distribution (in cM) for the genetic maps and the anchored genomic region (in Mbp) for the physical map, while the right panel is showing the Marey maps, *i.e.*, the correspondence between the physical (x-axis) and recombination-based (y-axis) position of markers. The female map is depicted in green and the male map is depicted in orange.

The parental framework maps were used to produce a physical map, Potra v1.2, of the *P. tremula* chromosomes that we used to estimate recombination maps (Figure 1, Figure S6). The scaffolds anchored from the parental framework maps spanned 210.7 Mbp and 205.1 Mbp for the female and male respectively. This corresponds to 54.6% and 53.1% of the 385.8 Mbp covered by the v1.1 assembly (Table S1). Of the 4,761 scaffolds with markers, 96.6% could be anchored in the assembly providing a total physical assembly consisting of 223.4 Mbp. This corresponds to 57.9% of the v1.1 *P. tremula* assembly (Lin *et al.* 2018) (Table 2). 75.7% of the physical map was both anchored and oriented, while the remaining 24.3% was only anchored. 199,967 of the v1.1 assembly scaffolds were not covered by the framework maps and thus could not be anchored to the physical map. There was a clear distinction between the scaffolds we could and could not anchor to the Potra v1.2 assembly. The median length for the 4214 scaffolds anchored in the map was 37 kbp and these scaffolds contain 26,808 predicted gene models. Conversely, the median length on unanchored scaffolds was only 0.3 kbp and they collectively contain only 8,501 predicted gene models (Table 2). After the initial assembly, gap estimation added 43 Mbp of gap sequences across the genome, increasing the estimated total size of the v1.2 assembly to 265 Mbp. This is approximately 55% of the 479 Mbp genome size estimated for *P. tremula* (Lin *et al.* 2018).

**Table 2:** Summary of physical assembly Potra v1.2

	Anchored	Oriented	Unplaced
Markers (unique)	19,302	15,597	215
Markers per Mbp	86.4	92.2	1.3
Gene elements	26,808 (75.9%)	NA	8501 (24.1%)
N50 Scaffolds (bp)	2,071	1,589	238
Scaffolds	4,599	2,759	200,129
Scaffolds with 1 marker	1,151	0	133
Scaffolds with 2 markers	999	787	17
Scaffolds with 3 markers	620	384	7
Scaffolds with >=4 markers	1,829	1,588	5
Total bases	223,381,844 (57,9%)	169,187,619 (43,9%)	162,436,408 (42,1%)

### Recombination estimates

**Figure 2:**
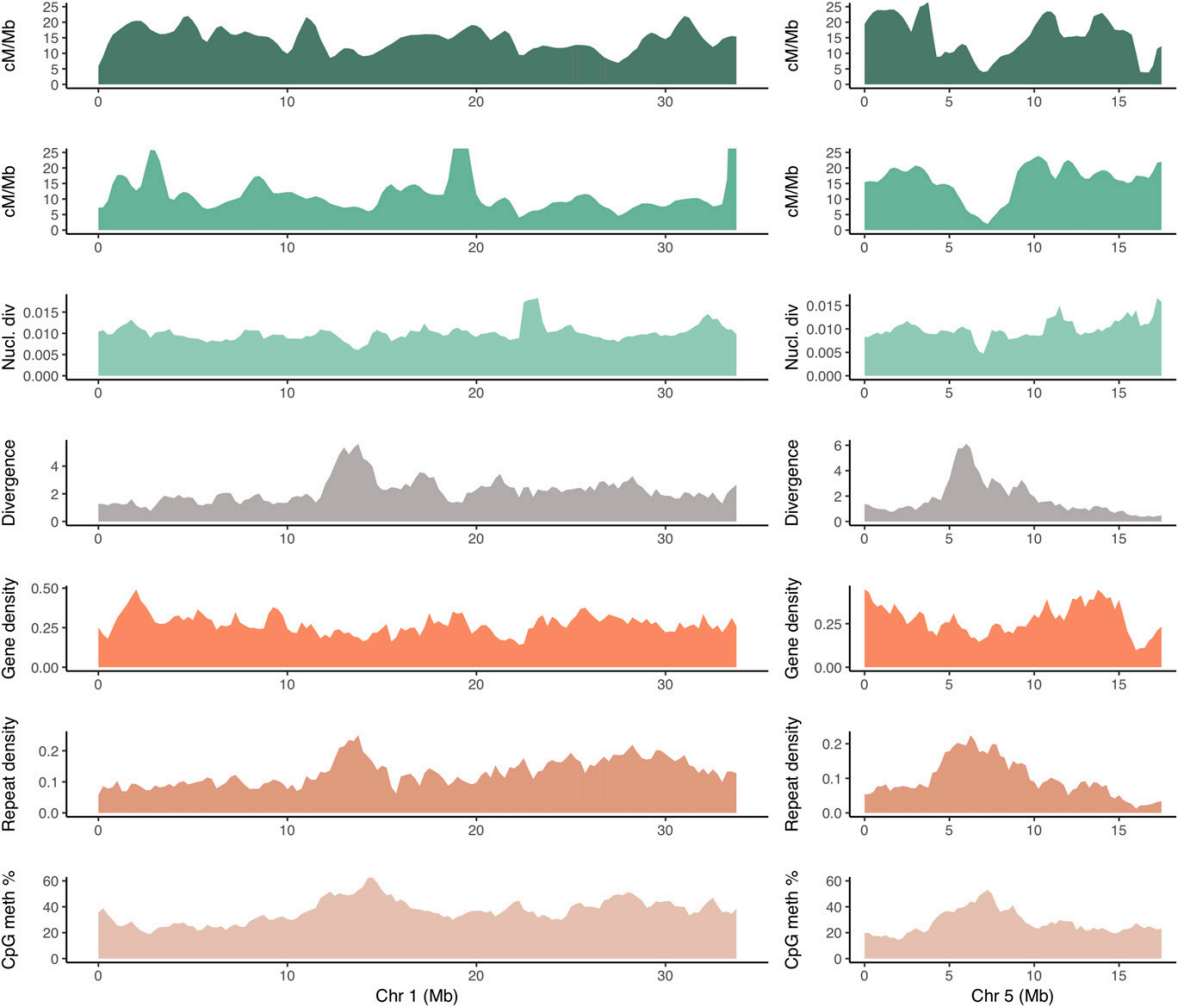
Recombination rates and genomic features calculated in 1 Mbp windows across chromosome 1 and 5 with a step size of 250kbp. A) Recombination rate estimated from the linkage map (cM/Mbp) B) Recombination rate estimated from sequence LD data (cM/Mbp) C) Nucleotide diversity (1/bp) D) Divergence (sites/Mbp) E) Gene density (percentage coding/Mbp) F) Repeat density (percentage repeats/Mbp) G) CpG methylation (percentage/Mbp).

Recombination estimates were produced based on the consensus linkage map (LMB) and from LD data (LDB) derived from 25 randomly selected individuals from the SwAsp collection. The LMB recombination rate estimates varied between 1.605 cM/Mbp on chromosome 4 to 26.911 cM/Mbp on chromosome 11, while the LDB estimates varied between 1.969 cM/Mbp on chromosome 5 to 231.801 cM/Mbp on chromosome 1. The median estimated recombination rate in the LMB map was 16.0 cM/Mbp with a mean of 15.6 cM/Mbp, whereas the median recombination rate for the LDB map was 14.0 cM/Mbp with a mean of 16.1 cM/Mbp (Table S2, Figure S7). The majority of all recombination rate estimates (97%) for both maps fell in the range of 2 - 27 cM/Mb (Figure 2, Figure S8). There were 20 windows where the LDB estimates are 1.5-15-fold higher compared to the corresponding rates from the LMB estimates and 2-14-fold higher than the mean recombination rate estimate from the LDB map. 13 of these windows had recombination rates exceeding 27 cM/Mbp, while seven were within 2-27 cM/Mbp. Conversely, there were also 23 windows where the LDB estimates were 2-4 times lower than the corresponding LMB estimates (Figure 2, Figures S8 and S9).

### Correlation of recombination rate estimates and genomic features

**Figure 3:**
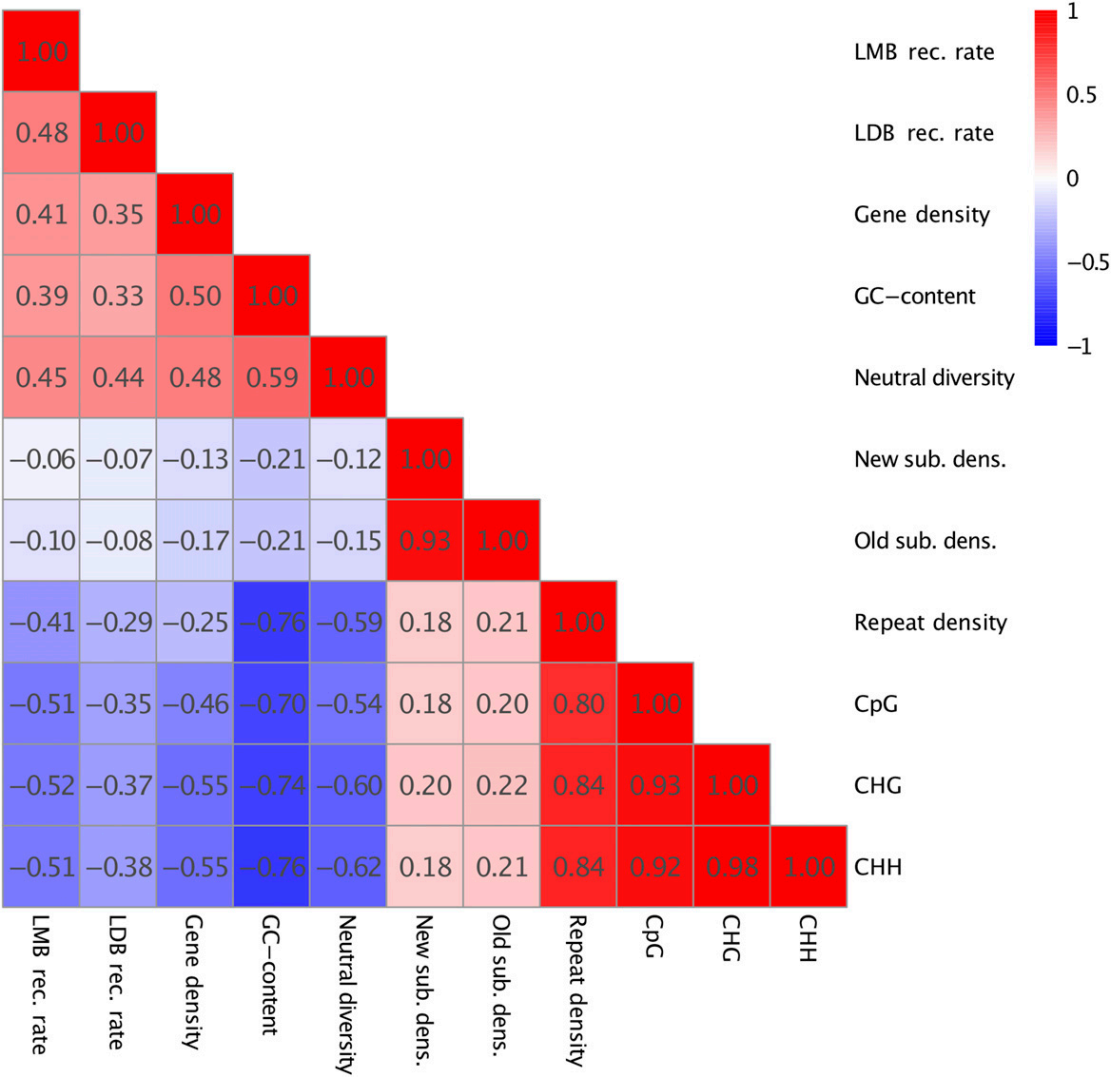
Correlations between recombination rates and genomic features. All correlations are significant (*P* < 0.01) with the exception of those marked with * (*P* > 0.01).

The Spearman’s rank correlation between recombination rate estimates for the LMB and LDB maps was 0.478 (Figure 3). This was the strongest positive correlation of all of the correlations calculated for both maps and strongest correlation overall for the LDB map. Correlations between the LMB and LDB recombination maps and neutral diversity were the second strongest positive correlations for both maps, 0.447 and 0.442 respectively. This correlation was the second strongest overall for LDB map. Correlation with neutral diversity was also the only variable where there was no notable decrease in the correlation coefficient from the LMB to the LDB recombination maps (Figure 3).

For the LMB map we observed strong negative correlations with CHG methylation (-0.515), CHH methylation (-0.511) and CpG methylation (-0.505). For the LDB map the corresponding correlations were -0.379 (CHH), -0.371 (CHG) and -0.353 (CpG). Methylation levels were also strongly correlated with each other (0.918-0.984) and with repeat density (0.800-0.840). Repeat density was also moderately negatively correlated with recombination rate estimates from both the LMB (-0.408) and LDB maps (-0.291). Both recombination maps showed only weak correlations (-0.1<*ρ*<0.1) with either old or new neutral substitution densities (-0.06 - -0.1). Neutral substitution densities and neutral diversity showed only weak negative correlations (Figure 3). Overall, the LMB estimates displayed consistently stronger correlations with the different genomic features compared to the LDB estimates. This is in line with 5% of the total variation being explained by a multiple regression model for the LDB recombination estimates compared to 35% variation explained for the LMB estimates (Table 3).

**Table 3:** Multiple regression of recombination rate and various genomic features

	Factor	Estimate	Std. Error	*t*	*p*
**Linkage map-based recombination rates**					
	Gene density	0.225	0.037	6.12	1.3e-9
	Repeat density	−0.111	0.069	−1.60	0.111
	GC content	−0.182	0.042	−4.37	1.36e-5
	GpG methylation	−0.360	0.050	−7.18	1.25e-12
	Nucleotide diversity	0.140	0.034	4.07	5.0e-5
	Substitution density	−0.038	0.056	−0.685	0.493
					*R^2^* = 0.345
**LD-based recombination rates**					
	Gene density	−0.009	0.031	−0.11	0.836
	Repeat density	0.218	0.083	2.63	8.6e-3
	GC content	0.111	0.050	2.22	0.027
	GpG methylation	−0.021	0.060	−0.36	0.717
	Nucleotide diversity	0.176	0.041	4.26	2.2e-5
	Substitution density	−0.163	0.067	−2.47	0.015
					*R^2^* = 0.054

## Discussion

### P. tremula fine-scale genetic maps and physical assembly Potra v1.2

The genetic maps presented here are the most marker-dense maps produced for *P. tremula* to date. Our female map is only 20 cM larger than the male map, despite having 518 more informative markers (Table 1) and most chromosomes have size differences of less than 10 cM between sexes (Table 1, Figure S6). However, in cases where we observed differences between the maps for the two sexes exceeding 10 cM, the male map is shorter in all but one case. These results, together with the overall shorter linkage map for the male, could suggest overall lower recombination rates in males, in line with what has been observed in many other highly outcrossing plant species (Lenormand and Dutheil 2005). The high marker density in our framework genetic maps allowed us to anchor 57.9% of the *P. tremula* v1.1 genome assembly on to the expected 19 chromosomes, providing us with the first chromosome-scale assembly for *P. tremula* (Table 2).

The map length in the section *Populus*, to which *P. tremula* belongs (Wang *et al.* 2014), has previously been estimated to be 1,600-3,500 cM (*e.g.*, Zhang *et al.* 2004, Paolucci *et al.* 2010, Zhigunov *et al.* 2017). The most relevant comparison for our purposes is the recently produced linkage maps in *P. tremula* by Zhigunov *et al.* (2017). Their map contains 2000 informative markers with an average marker distance of 1.5 cM that were observed in 122 progenies, resulting in a total map length of 3000-3100 cM. Our framework maps are much denser with ca. 12000-13000 informative markers (Table 1) and with an average distance of ∼0.3 cM between markers. In addition, our map is based on a mapping population consisting of 764 progenies and we are hence able to achieve a far greater resolution in our maps. However, larger data sets, both with respect to the number of markers and the number of progenies used, increase the risk of genotyping errors. Genotyping errors will ultimately lead to an inflation of map sizes as errors can be interpreted as recombination events during map creation and this could help explain why our maps are roughly 1000 cM longer than those reported by Zhigunov *et al.* (2017), given that we use 5-7 -fold more markers and a mapping population that is six times larger.

On the other hand, our framework genetic maps are similar in size to the ca. 4200 cM and 3800 cM maps presented by Tong *et al.* (2016) for the more distantly related species *Populus deltoides* and *Populus simonii*, respectively. The large size of these maps led Tong *et al.* (2016) to suggest that their maps were suffering from inflation due to the difficulty of properly ordering a large number of markers within a linkage group. While we likely also suffer from such size inflation, these issues appear to be less severe in our *P. tremula* parental maps, which contain between 8-14 times the number of markers used in the *P. deltoides* (1601) and *P. simonii* (940) maps and yet yield linkage maps of similar size. One explanation for this is our considerably larger mapping population compared to the *P. deltoides* and *P. simonii* maps (299 progenies). A greater number of segregating progenies helps mitigate the problems of ordering a larger number of markers by increasing resolution of recombination detection.

### Recombination rate estimates

Recombination rates estimated from both the consensus linkage map and from polymorphism data showed substantial variation across all chromosomes on Mbp scales (Figure 2). For the consensus genetic map-based estimates and LD-based estimates, the majority of our observations fell in the range 0-27 cM/Mbp (Figure 2, Figure S8) which is similar to what has been observed in other plants such as *Arabidopsis thaliana* (Giraut *et al.* 2011), *Populus trichocarpa* (Slavov *et al.* 2012) and *Eucalyptus grandis* (Silva-Junior and Grattapaglia 2015), where recombination rates across chromosomes mostly fall within 0-25 cM/Mbp.

We observed a small number of genomic windows where the LDB recombination rates were either 1.5-15-fold higher or lower than the corresponding estimates based on the consensus genetic map (Figure 2, Figure S8). While we do not know for certain what causes these large differences in recombination rates, a possible explanation could be that such windows harbor recombination hotspots or coldspots that the comparatively coarse linkage map fails to detect. Recombination hotspots, with local recombination rates 10 to 100-fold higher than the genome-wide average, have been observed in a number of species, including *Drosophila melanogaster* (Chan *et al.* 2012), *Arabidopsis thaliana* (Kim *et al.* 2007), *Zea mays* (He and Dooner 2009), *Oryza sativa* (Si *et al.* 2015) and *Eucalyptus grandis* (Silva-Junior and Grattapaglia 2015). Similarly, coldspots have been identified in *Zea mays* (He and Dooner 2009) and *Oryza sativa* (Si *et al.* 2015) among others. Hotspots or coldspots for recombination are, however, often quite restricted in size (Choi and Henderson 2015), spanning only a few kbp, and the relatively coarse recombination maps produced here are consequently not suitable for accurate detection of such regions. It is also worth noting that in species with lower levels of diversity, the resolution of LD based estimates would be lower and the power to detect hot- and coldspots for LDB and LMB estimates may not differ much for these species.

The average recombination rate in *P. tremula* is 2-27 times higher than those found in a number of, mostly domesticated, plant and animal species (reviewed in Henderson (2012) and Tiley and Burleigh (2015)), suggesting that *P. tremula* exhibits recombination rates that are among the highest recorded in the animal and plant kingdoms. Of the species covered in these reviews, *P. trichocarpa* (Slavov *et al.* 2012) makes for the most interesting comparison since it is one of the few undomesticated species listed, is closely related to *P. tremula* and has previously been compared with *P. tremula* (Wang *et al.* 2016). Despite the close relationship, the average recombination rate in *P. trichocarpa* (Slavov *et al.* 2012) is less than a third of what we estimated for *P. tremula*. Similar observations were previously made by Wang *et al.* (2016) who found that population-based recombination rates in *P. trichocarpa* were on average only a quarter of the corresponding values in *P. tremula*. Wang *et al.* (2016) argued that the differences they observed in recombination rates between *P. tremula* and *P. trichocarpa* could at least partly stem from differences in the effective population size (N_e_) of the two species (Wang *et al.* 2016). In light of this, it would be interesting to perform further comparisons of recombination rates between *P. tremula* and other *Populus* species that have wide distribution ranges and large N_e_, such as *P. deltoides* (Tong *et al.* 2016) or *P. tremuloides* (Wang *et al.* 2016).

### Correlations between recombination rate and genomic features

Recombination rate estimates from the consensus linkage map and from polymorphism data showed a moderately strong positive correlation (>0.4) (Figure 3). A similar correlation between linkage map and LD-based estimates of recombination was also observed in *Mus musculus* by Booker *et al.* (2017), suggesting that LDB recombination rate estimates are reliable substitutes for genetic map-based recombination rate estimates. A recent study in *Gasterosteus aculeatus* (Shanfelter *et al.* 2019), however, found even stronger correlations between the two estimates (∼0.8).

We observed a strong positive correlation between recombination rate and gene density (0.45 and 0.41 respectively) (Figure 3). This is in line with earlier observations in plants (Tiley and Burleigh 2015, Stapley *et al.* 2017) and implies that recombination may be linked to gene-dense regions through a higher recruitment of the recombination machinery to euchromatic genome regions. Preferential recruitment of recombination to euchromatic genome regions has also been put forward as an explanation for why recombination rates across plants generally show stronger correlations with gene density compared to genome size (Henderson 2012, Tiley and Burleigh 2015). Studies in plants like *Arabidopsis thaliana* and *Oryza sativa* have shown that while crossover events are enriched in genic regions, they mostly occur in promoters a few hundred bps upstream of the transcription start site or downstream of the transcription termination site (Choi *et al.* 2013, Marand *et al.* 2019).

We observed negative correlations between local recombination rates and both repeat density and methylation (Figure 3), in line with earlier results that highlighted the role of chromatin features in establishing crossover locations in plants (Choi *et al.* 2013, Marand *et al.* 2019). For instance, Choi *et al.* (2013) showed that methylation is lower at observed sites of crossovers and Rodgers-Melnick *et al.* (2015) showed that cross-over density in *Zea mays* is negatively correlated with repeats and CpG methylation. All methylation contexts were highly correlated in our data and also strongly correlated with repeat density (≥0.8, Figure 3), in line with the observation that most repetitive elements in plant genomes are strongly methylated (Saze and Kakutani 2011).

Compared to earlier results from *P. tremula*, we observed a weaker correlation between recombination rates and gene density (Wang *et al.* 2016). One possible reason for this is likely to be the reference genome used. Wang *et al.* (2016) based their analyses on the *P. trichocarpa* reference genome whereas our analyses were based on a *de novo* assembly for *P. tremula*. The *P. trichocarpa* assembly, while more contiguous than our current *P. tremula* assembly, is less ideal for these types of analyses since divergence between the two species leads to substantially reduced rates of read mapping primarily in intergenic regions (Lin *et al.* 2018). Our current assembly, while only representing 55% of the expected genome size *of P. tremula*, likely offers a more unbiased set of genomic regions where we are able to call genetic variants. In contrast, the data derived from using the *P. trichocarpa* reference genome likely suffers from under-representations of repeat-rich regions and other intergenic regions (Wang *et al.* 2016).

GC-content was positively correlated with both our recombination rate estimates, similar to what has been observed in humans (*Homo sapiens*) (*e.g.*, Fullerton *et al.* 2001) and *Arabidopsis thaliana* (Kim *et al.* 2007) among others. However, when GC-content was included in a multiple regression model with other genomic features, the direct effect of GC content was actually negative for the LMB recombination rate (Table 3). GC-content is strongly correlated with gene density (0.50) in *P. tremula*, and gene density is in turn also strongly positively correlated with recombination (Figure 3). The strand separation needed in the strand invasion of meiotic recombination is harder to achieve in areas with high GC-content due to higher annealing energy and can explain why GC-content has a direct negative effect on recombination rates when effects of gene density are accounted for (Table 3, *e.g.*, Mandel and Marmur 1968).

### Effects of linked selection on patterns of nucleotide diversity in P. tremula

Both of our recombination rate estimates were strongly correlated with nucleotide diversity at putatively neutral sites (Figure 3). A positive correlation between local recombination rate and nucleotide polymorphism is usually interpreted as a signature of ubiquitous natural selection acting either through positive (hitchhiking) or negative (background) selection (Begun and Aquadro 1992). Alternatively, such a correlation could also arise if recombination itself is mutagenic (Begun and Aquadro 1992). However, if recombination is mutagenic one also expects to see a correlation between recombination and sequence divergence at neutral sites (Begun and Aquadro 1992). Our data show little evidence supporting the idea that recombination has a direct mutagenic effect as we observed only a weak and negative correlation between local recombination rates and substitutions at putatively neutral sites (Figure 3). In light of this, and in line with earlier results, we observed that linked selection has pervasive effects on neutral diversity across the *P. tremula* genome (Ingvarsson 2010, Wang *et al.* 2016).

### Conclusions

Our high-density *Populus tremula* genetic maps and the new chromosome-scale genome assembly we present here provide a valuable resource not only for *P. tremula*, but also for comparative genomics studies within the entire genus *Populus*. Estimates of recombination rates derived from two different approaches were in broad agreement and showed similar correlations various genomic features. However, our results suggest that LD-based estimates of recombination might be especially useful for identifying fine scale recombination variation and for identifying features such as recombination hot- or cold-spots as it is based on information derived from population-scale variation. We have also verified and extended the observation that linked selection is an important force shaping genome-wide variation in *P. tremula* by showing that the positive correlation between local recombination rates and nucleotide diversity and neutral sites is robust even when factoring in the effects of other genomic features. Although a positive correlation between recombination and diversity is a hallmark signature of linked selection, the pattern can be established by either positive or negative selection. We have earlier documented evidence for both a reduction in levels of standing variation due to recurrent hitchhiking (Ingvarsson 2010) and a reduction in the efficacy of purifying selection at eliminating weakly deleterious variants in regions of low recombination (Wang *et al.* 2016). More work is thus needed to assess the relative importance of positive and negative selection in shaping genome-wide variation in *P. tremula* and having access to the resources developed here will facilitate these studies.
